# Comparative Study on the Efficacy of Anorganic Bovine Bone (Bio-Oss) and Nanocrystalline Hydroxyapatite (Ostim) in Maxillary Sinus Floor Augmentation

**DOI:** 10.1155/2014/967091

**Published:** 2014-10-29

**Authors:** Adileh Shirmohammadi, Leila Roshangar, Mohammad Taghi Chitsazi, Reza Pourabbas, Masoumeh Faramarzie, Nasrin Rahmanpour

**Affiliations:** ^1^Dental and Periodontal Research Center, Tabriz University of Medical Sciences, Tabriz 5166614711, Iran; ^2^Department of Anatomy and Histology, Faculty of Medicine, Tabriz University of Medical Sciences, Tabriz 5166614711, Iran; ^3^Department of Periodontics, Tabriz Dental Faculty, Tabriz 5166614711, Iran

## Abstract

*Purpose*. The aim of this study was to evaluate the efficacy of anorganic bovine bone (Bio-Oss) in comparison with nanocrystalline hydroxyapatite (Ostim) in sinus floor augmentation. *Methods*. Ten patients aged 40–80 were selected. All the patients needed sinus floor augmentation due to insufficient bone for simultaneous implant placement. The patients underwent panoramic radiography and cone beam computed tomography (CBCT) prior to surgical procedure. After lifting the sinus membrane, Bio-Oss and Ostim are randomly grafted at one of the two sides. Biopsies were obtained from areas identified 5 months after the surgery and before implant placement and then were prepared for histological analysis. Statistical analysis was performed with nonparametric Wilcoxon signed-rank test for comparison of histological and radiological parameters between the two groups. *Results*. Histological findings revealed a significant increase in percentages of new bone in the Ostim group (*P* = 0.015). Furthermore, new bone density was greater with Ostim compared to Bio-Oss (*P* = 0.038); however, the difference in height increase after surgery did not reach statistical significance (*P* = 0.191). *Conclusion*. Despite the limitations of this trial, Ostim and Bio-Oss are useful biomaterials in sinus augmentation and Ostim seems to be even more effective in new bone formation.

## 1. Introduction

Application of implant in the posterior maxilla often poses multiple therapeutic challenges due to the atrophy of the alveolar ridge and sinus pneumatization. Proximity of maxillary sinus floor to the alveolar ridge leads to a reduction of the height and bone volume and therefore the implant placement with appropriate length and dimension is limited [1–3]. To overcome this, different surgical procedures, such as sinus floor augmentation, have been suggested. This procedure is a predictable treatment option due to an increase in vertical dimension and quantity of bone in the posterior maxilla. Thus, it is possible to rehabilitate jaw by placing implants in this region [4].

The lateral window technique is a common method. This procedure was introduced in 1980 by Boyne and James [5]. It provides a window in the lateral wall of the sinus; by carefully and gently lifting the sinus membrane, bone* graft *is utilized in the space created [6]. Autogenous bone graft has been proposed as the gold standard for sinus procedure with good long-term results. This is due to the fact that autogenous bone is osteogenic, osteoinductive, and osteoconductive without the risk of graft rejection or adverse reactions [7]. However, these materials have disadvantages, such as limited availability, limited bone volume, unpredictable resorption, need for a second surgical site, and donor site morbidity. Therefore, to resolve these problems alternative materials with different features have been developed [8, 9]. Xenografts and synthetic biomaterials are used for hard tissue augmentation. They contain no living cells to induce osteogenesis and just serve as a scaffold with a prolonged healing time [10].

Allografts are prepared from hard tissues of the same species with unequal genotypes. Their main feature is osteoconduction. However, allografts are osteoinductive under some conditions [11]. Although these grafts have advantages such as availability of infinite amounts of materials and no donor site morbidity, their use is restricted because of the risk of infectious disease transmission, host immune reaction, and lower predictability of outcomes in sinus procedures [12].

Xenografts are harvested from different species. Most studies have shown that the resorption of their particles is slow. It is thought that this feature is desirable for stability and maintenance of volume in sinus augmentation [11, 13]. Bio-Oss is a biocompatible material with high potential for increasing new bone formation. However, in some studies delayed healing has been observed in graft region with this material [14, 15].

Disadvantages of these biomaterials include the potential of disease transmission and reaction of the host immune system. In addition, some patients might refuse use of animal materials such as Bio-Oss [15].

Due to disadvantages and limitations of allografts and xenografts, more attention has been focused on alloplastic materials. It is known that alloplastic materials act as a scaffold with benefits such as availability, ease of manipulation, fewer complications, and high security [16].

Recently a synthetic nanocrystalline hydroxyapatite (Ostim) has been proposed as a suitable material for hard tissue augmentation. Its chemical composition is almost the same as that of bone mineral. Furthermore, replacement by bone is facilitated due to very fine particles (18 nm) [17]. One study indicated that bone formation can be promoted by this material in intrabony defects [18].

The favorable results of studies on Ostim encouraged us to use this material for sinus augmentation procedures.

To date, a wide range of alloplastic materials have been studied for sinus augmentation. Nevertheless, to our knowledge no research studies have been performed on sinus procedures in order to compare Ostim and Bio-Oss. The present clinical trial was designed to evaluate whether Ostim can be a better choice than Bio-Oss in sinus augmentation. Percentage of new bone formation 5 months after sinus augmentation was defined as the primary outcome.

## 2. Materials and Methods

### 2.1. Patient Selection

This study was designed as a double-blind randomized clinical trial with controlled split-mouth design. In the present study 10 patients (8 males and 2 females), aged 40 to 80 (mean age of 54 years), were selected from patients referring to Implant Department of Tabriz Faculty of Dentistry from April 2012 to January 2013. All the patients were partially or completely edentulous in the posterior maxilla and needed sinus augmentation due to advanced vertical bone loss and insufficient bone for simultanous implant placement. All the alternative treatment modalities were explained in detail to patients and all the patients chose sinus augmentation surgery.

All the patients were in good health with no medical problems. They were informed about the nature of the study and surgical procedures and then they signed a consent form.

The study protocol was approved by the Research Ethics Committee of Tabriz University of Medical Sciences after a review by Dental and Periodontal Research Center of Tabriz University of Medical Sciences. This study was registered in Iranian Registry of Clinical Trials (IRCT) and allocated the code IRCT201204157128N2.

The inclusion criteria of the trial were as follows:

- severe atrophy in the posterior maxilla;
- residual bone height of less than 5 mm between the sinus floor and the alveolar crest.

The exclusion criteria of the study were plaque index and bleeding index of >25%, smoking, pregnancy, periodontal disease, and periapical lesions in adjoining teeth, maxillary sinus pathologic lesions, and systemic diseases (e.g., uncontrolled diabetes, heart diseases, and blood disorders) and metabolic disorders (e.g., osteoporosis and hyperparathyroidism), radiotherapy of the head and neck due to malignancy, bisphosphonate and immunosuppressive drugs, and any condition affecting the hard and soft tissue healing process.

### 2.2. Surgical Protocol and Medication

All the patients underwent digital panoramic radiography and cone beam computed tomography (CBCT) scan prior to the surgical procedure. For each patient phase I periodontal therapy was performed 2-3 weeks before surgery. They received antibiotics (1 gr amoxycillin and clavulanic acid) one day prior to surgery and continued for 5–7 days as ordered.

All the patients used 0.2% chlorhexidine gluconate for 2 minutes before surgery.

In this clinical trial a lateral window technique was applied for all the patients. After local infiltration anesthesia (2% lidocaine with epinephrine 1 : 100,000), a mucoperiosteal flap was reflected via a horizontal midcrestal incision and a vertical releasing incision. Then an access window was prepared into the lateral sinus wall with a round diamond bur.

After preparation of the lateral window, the sinus floor membrane was separated from the walls of bone inside the sinus by careful attention to minimize its perforation and maintain its integrity.

**Figure 1:**
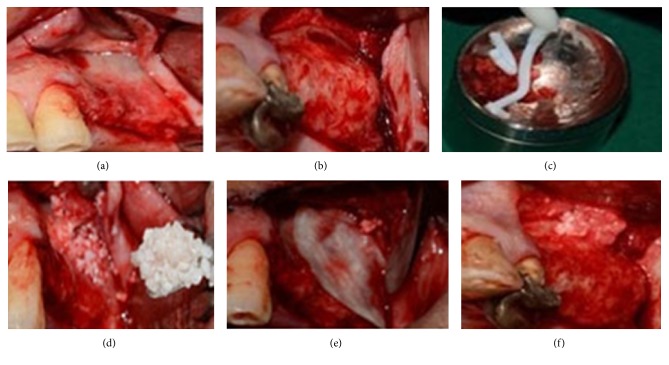
Sinus augmentation procedure with Bio-Oss: (a) flap reflection and clinical view of lateral wall after elevation of the flap; (b) buccal window in the sinus after flap reflection and elevation of membrane; (c) Ostim mixed with autograft: (d), (e) sinus filled with Bio-Oss and autograft; (f) the lateral wall covered with a collagen membrane.

After elevation of the sinus membrane on a random basis, on one side, Ostim (Heraeus Kulzer GmbH and 63450 Hanau, Germany) with 20% autogenous bone graft was used and on the other side Bio-Oss (Geistlich Pharma AG and 6110 Wolhusen, Switzerland) with 20% autogenous bone graft was applied. Autogenous bone graft was provided from maxillary tuberosity and other available sites. Subsequently a collagen membrane (Iranian Tissue Bank Research & Preparation Center, Tehran) was applied on the access window. The flaps were sutured with 4.0 nonabsorbable surgical sutures and then the patients received postoperative medical instructions (Figure 1).

**Figure 2:**
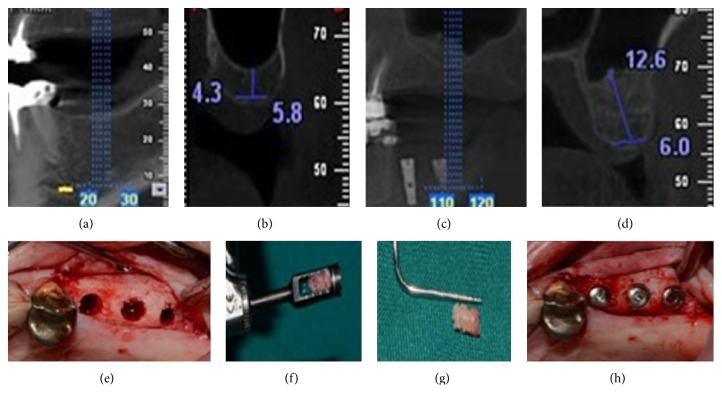
(a), (b), (c), (d) cross-sectional images of grafted side before and after sinus augmentation with Ostim; (e) flap reflection in buccal view and the clinical view of osteotomy sites; (f), (g) the biopsy specimens taken by trephine bur with an internal diameter of 2 mm; (h) insertion of implants into # 25, # 26, and # 27 sites.

In addition, the patients were advised not to use the prosthesis during the first 2 weeks of healing period. Follow-up CBCT scans for each patient were ordered immediately after surgery and at the time of implant insertion (Figure 2).

### 2.3. Second Surgery and Histological Preparation

**Figure 3:**
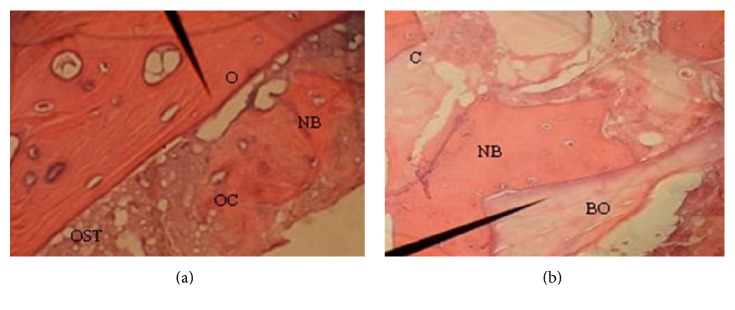
Histological images of Ostim (a) and Bio-Oss (b) specimens retrieved 5 months after surgery. Arrow in A shows osteoid (O) in the margin and new bone (NB) inside Ostim particles (OST) and osteocytes (OC) within lacuna and connective tissue (C). This image indicated signs of active bone remodeling and resorption with Ostim. Arrow in (b) shows particles of the Bio-Oss (BO), new bone (NB) around the Bio-Oss particles, and connective tissue (C) (hematoxylin and eosin; ×20).

Biopsies were collected 5 months after the sinus procedure prior to implant insertion using a trephine bur with an internal diameter of 2 mm. Biopsy specimens were coded in bottles containing 10% buffered formalin, with a pH value of 7 and were sent for histological evaluation (Figure 2). The histologist (LR) was blinded to the type of the bone grafts used in sinus augmentation. Subsequently, the osteotomy sites were corrected by drilling and implants with appropriate lengths were placed. Biopsy specimens were fixed in 10% buffered formalin for 10 days and then decalcified with 65% nitric acid for 72 hours. The decalcified samples were evaluated every day. They were fixed in 10% buffered formalin again for a week and then rinsed in running water. The specimens were dehydrated by increasing concentrations of ethanol for an hour and then washed twice with xylene. They were embedded in paraffin and then cut to a thickness of 5-6 micrometer by a microtome. The specimens were stained with hematoxylin and eosin (H&E) using standard technique and prepared for histological analysis. The parameters evaluated were the percentage of new bone formation, residual bone grafts, and connective tissue under a light microscope (BX40, Olympus, Germany) and a system of the image analysis with the help of a computer (Motic software image, Figure 3).

To measure and evaluate bone obtained before and after surgery, panoramic radiography and CBCT were used. Bone height was determined from the ridge crest to the floor of the sinus before augmentation and 5 months later. New bone density in the operation site was obtained by NNT Viewer software, version 2.21. Measurements were calculated by an examiner (A Sh) who was blinded to the surgical procedure.

### 2.4. Analysis of Data

The median and interquartile ranges (IQR) were used to describe the samples. For statistical analysis, the nonparametric Wilcoxon signed-rank test was applied in order to compare the histological and radiological parameters between the two groups. A *P* value of <0.05 was considered statistically significant.

## 3. Results

A total of 10 patients (8 males and 2 females), aged 40 to 80 (mean age of 54 years), were enrolled in this study and bilateral sinus augmentation was performed for all the patients. In 9 of 10 patients, complications such as infection, inflammatory reactions, pain, and wound dehiscence did not appear during the healing period. None of the membranes were perforated during surgery.

In one patient sinus infection occurred at Ostim site. Therefore, this patient was excluded from the study because the study design was split-mouth. Therefore data were analyzed for 9 patients in whom bilateral biopsies had been removed 5 months after surgery. Apart from the patient mentioned above, the designed implants were placed in the augmented area, all with primary stability. In addition, in the clinical examination, from the point of view of density, lateral window was tightly closed in the Ostim group compared with the Bio-Oss group.

### 3.1. Histological Evaluation and Histomorphometric Data

Biopsies were collected from the designated sites 5 months after sinus augmentation. Histological evaluation of Ostim specimens revealed osteocytes within lacuna in the regenerated bone. Unmineralized osteoids were found around the resorbing biomaterials. The residual particles of Ostim were seen in some areas in close contact with the new bone (NB). In addition, the autograft particles with active bone resorption were seen. Inflammatory cell infiltration and foreign body reaction were not observed in these specimens.

Histological evaluation of Bio-Oss specimens revealed NB around Bio-Oss particles (direct bone contact). The residual Bio-Oss was surrounded by NB and in some areas it was seen in close contact with NB. In addition, interparticle bridging was observed in Bio-Oss graft. Inflammatory cell infiltration and foreign body reaction were not seen in Bio-Oss specimens.

Resorption of Bio-Oss particles was observed at lower levels, while in Ostim and autogenous bone graft, the process of the active resorption and replacement with NB were seen.

Histomorphometric data of 18 specimens revealed more NB with normal bone remodeling in the Ostim group compared with the Bio-Oss group (Table 1). Median percentages of NB in the Ostim and Bio-Oss groups were 25.3% and 21.9%, respectively. In addition, percentage of residual materials in the Bio-Oss group was greater than that in the Ostim group: 33.13% versus 20.8%, respectively. There were no differences in connective tissues between the two groups (*P* = 0.173).

### 3.2. Radiological Findings

In order to measure and evaluate bone obtained before and after surgery panoramic radiographs and CBCT scans were used. Bone height was determined from the ridge crest to the floor of the sinus before augmentation at baseline and 5 months later. Comparison of radiographic findings indicated that new bone density was significantly different in Ostim and Bio-Oss groups, with 317 and 294 Hounsfield units, respectively (*P* = 0.038); however, the difference in augmentation height did not reach statistical significance (*P* = 0.191).

Comparison of bone height at baseline and 5 months after surgery revealed a significant increase in the median of bone height in both groups. In the Ostim group, the median of bone height increased from 3.2 mm to 11.4 mm (*P* = 0.007) and in the Bio-Oss group it increased from 3.7 mm to 11.1 mm (*P* = 0.008).

Table 1 shows histomorphometric data, including the percentage of new bone, residual materials, and connective tissue in the test (Ostim) and control group (Bio-Oss) and radiographic findings of new bone density and augmentation height with CBCT.

## 4. Discussion

The present trial was designed to compare the efficacy of Bio-Oss and Ostim in sinus augmentation in relation to new bone formation and its height and density 5 months after sinus augmentation. Current advances in sinus augmentation with the use of bone grafts have resulted in great developments in the field of reconstruction of edentulous jaws and improvement of quality of life, although the majority of research studies have yielded variable and different outcomes [9, 19].

It has been established that chemical structure of biomaterials can determine the type of tissue that will grow into its porous matrix and also the amount of new bone formation in sinus augmentation [20]. This issue was clearly demonstrated in the present study. Due to differences in the characteristic of Ostim and Bio-Oss the results obtained in this study were different.

Bio-Oss is a deproteinized sterilized bovine bone and exhibits chemical and physical characteristics similar to human bone. Highly porous structure and a great contact surface area of this material promote capillary ingrowth and migration and proliferation of osteoblasts [15, 21].

On the other hand, Ostim is a pure synthetic material and its chemical structure is similar to the bone mineral composition. Ostim serves as a scaffold to accelerate new vascularization in grafted area. Particle size of Ostim in the nanometer range leads to a significant increase in contact surface area and promotes new bone regeneration [17, 18].

In this study 20% autogenous bone graft was applied with 80% Bio-Oss or Ostim based on the findings of studies by Hallman and Rickert [10, 22]. The results of these studies showed an increase in bone regeneration when a mixture of autograft and Bio-Oss was used, although well-designed studies should be performed for more clarification. In addition, it has been suggested that Ostim should be mixed with a stable material due to lack of dimensional stability [23]. In this study, it was easy to work with Ostim when mixed with autogenous bone.

Biopsies were taken 5 months after surgery because in most studies a sampling time of 5-6 months has been proposed [10].

In the present trial median percentages of new bone formation in the Ostim and Bio-Oss groups were 25.3% and 21.9%, respectively. In relation to Bio-Oss, the findings of this study were consistent with other studies [24, 25]. These studies have indicated new bone formation percentage rates of 42.1 ± 10% to 14.7 ± 5% at 4-to-12-month intervals with the use of Bio-Oss for sinus augmentation, although it is assumed that these results were different at different intervals.

Since the rate of new bone formation is an important index of sinus augmentation [26], increased new bone in the Ostim group can be very noticeable. This might be attributed to very fine particles of Ostim, which can lead to a significant increase in contact surface area. However, much information is not available regarding Ostim and, to our knowledge, there is no investigation comparing the efficacy of this material with that of Bio-Oss in sinus augmentation. As mentioned previously the histological findings of this study revealed a significant increase in percentage of new bone in the Ostim group (*P* = 0.015). These findings were similar to the results of various research studies which have confirmed high rates of dense cortical and cancellous bone ingrowth into bony defects with the use of Ostim [15, 16].

Percentages of residual materials in the Bio-Oss group were greater than those in the Ostim group: 33.13% versus 20.8%, respectively, in this study. This finding shows that the amount of new bone is lower than the amount of residual particles of Bio-Oss, whereas in the Ostim group the amount of new bone was higher than the amount of residual particles. Whether these residual particles are required after a certain time for maintaining the stability of the graft is still not well understood and further research is necessary.

Panoramic radiographs and CBCT were used to measure and evaluate bone obtained before and after surgery. It has been shown that changes in the height of the graft material can accurately be diagnosed by three-dimensional radiography [27].

Comparison of bone height at baseline and 5 months after surgery by radiography revealed a significant increase in the median of bone height in both groups. In the Ostim group, the median of bone height increased from 3.2 to 11.4 mm (*P* = 0.007) and in the Bio-Oss group it increased from 3.7 to 11.1 mm (*P* = 0.008), indicating that Ostim is an effective material for sinus augmentation, similar to Bio-Oss.

Despite the fact that bone density was higher in the Ostim group compared to the Bio-Oss group, the quality of bone was D4 in both and therefore it was not clinically significant.

In addition, from the point of view of density, lateral window was tightly closed in the Ostim group compared with the Bio-Oss group.

10 patients aged 40–80 were selected in this study. Because the age range is wide, it may influence the results, but Wolf et al. investigated whether the patient's age has an effect on bone formation and incorporation in maxillary sinus floor augmentation procedures (the fully synthetic nanocrystalline bone augmentation material used in this study) and could not show an age-dependent difference in investigated parameters between the age groups [28].

Limitations of the present study were the small sample size and short follow-up time.

Considering the importance of this issue, further studies with larger sample sizes and longer follow-up periods of these patients are recommended for more thorough evaluations.

## 5. Conclusion

Despite the limitations of this clinical trial, Ostim and Bio-Oss are useful biomaterials in sinus augmentation and Ostim seems to be even more effective.

The results of this study suggest that Ostim probably has the potential to produce more new bone in a short time after sinus augmentation. However, further research studies with more focus on the current topic are recommended.

## Figures and Tables

**Table 1:** Results of histomorphometric and radiographic findings by Bio-Oss and Ostim biomaterials.

Patient number	Histomorphometric findings						Radiological findings			
	Percentage of residual materials		Percentage of new bone		Percentage of connective tissue		New bone density (hounsfield units)		Augmentation height (mm)	
	Bio-Oss	Ostim	Bio-Oss	Ostim	Bio-Oss	Ostim	Bio-Oss	Ostim	Bio-Oss	Ostim
1	14.70	15.90	22.10	24.20	63.20	59.90	301.00	290.00	7.10	7.90
2	25.00	28.10	20.23	32.70	54.77	39.20	285.00	322.00	7.10	10.20
3	41.05	27.30	12.75	21.20	46.20	51.50	295.00	334.00	8.30	7.90
4	36.50	23.30	12.30	18.20	51.20	58.50	229.00	254.00	7.30	7.60
5	39.90	20.80	26.10	25.30	34.00	53.90	299.00	339.00	7.50	8.20
6	28.40	16.90	13.16	21.30	58.44	61.80	313.00	292.00	7.20	6.90
7	33.13	21.70	26.83	34.20	40.04	44.10	261.00	317.00	7.40	8.30
8	28.90	19.20	21.90	33.50	49.20	47.30	279.00	286.00	7.60	7.90
9	33.40	18.10	27.63	28.10	38.97	53.80	294.00	320.00	8.30	7.60
Median	33.13	20.80	21.9	25.3	49.2	53.8	294	317	7.4	7.9
Median of differences (IQR)	−11.50 (−14.52–−11.50)		7.37 (1.28–10.02)		4.06 (−2.60–11.06)		26.00 (−2.00–39.50)		0.30 (−0.35–0.85)	
*P* value	0.021		0.015		0.173		0.038		0.191	
