# Corrigendum to “Comparative Study on the Efficacy of Anorganic Bovine Bone (Bio-Oss) and Nanocrystalline Hydroxyapatite (Ostim) in Maxillary Sinus Floor Augmentation”

**DOI:** 10.1155/2017/7258513

**Published:** 2017-05-11

**Authors:** Adileh Shirmohammadi, Leila Roshangar, Mohammad Taghi Chitsazi, Reza Pourabbas, Masoumeh Faramarzi, Nasrin Rahmanpour

**Affiliations:** ^1^Dental and Periodontal Research Center, Tabriz University of Medical Sciences, Tabriz 5166614711, Iran; ^2^Department of Anatomy and Histology, Faculty of Medicine, Tabriz University of Medical Sciences, Tabriz 5166614711, Iran; ^3^Department of Periodontics, Tabriz Dental Faculty, Tabriz 5166614711, Iran

In the article titled “Comparative Study on the Efficacy of Anorganic Bovine Bone (Bio-Oss) and Nanocrystalline Hydroxyapatite (Ostim) in Maxillary Sinus Floor Augmentation” [1], the last name of the fifth author was given incorrectly as “Faramarzie.” The author's name should have been written as “Faramarzi.” The revised authors' list is shown above.
